# Development of a Wire-Driven Robotic Fish Based on Double Sine Mechanism

**DOI:** 10.3390/biomimetics10030136

**Published:** 2025-02-24

**Authors:** Qian Yang, Qixin Wang, Zihao Cao, Zeyue Zhao, Ye Chen, Yong Zhong

**Affiliations:** Shien-Ming Wu School of Intelligent Engineering, Guangzhou International Campus, South China University of Technology, Guangzhou 510640, China; 202320160054@mail.scut.edu.cn (Q.Y.);

**Keywords:** robotic fish, wire-driven, double sine mechanism, different tail stiffness, various swimming frequencies

## Abstract

Wire-driven robotic fish can effectively simulate the movement of real fish, but research on high-frequency wire-driven robotic fish is limited. This paper introduces the development of wire-driven robotic fish based on a double-sine mechanism. The appearance of the fish body is designed based on the morphology of tuna, and a mechanism that can support the high-frequency movement of the wire-driven mechanism is designed. The swimming speed and turning performance of the robotic fish are experimentally tested at various swing frequencies. The experimental results show that within the range of 1 to 4 Hz, the swimming speed of the robotic fish with different tail stiffness increases as the frequency increases. However, when the frequency exceeds 4 Hz, the swimming speed decreases. The tail joint with lower stiffness performs better at low frequencies, but as frequency increases, higher stiffness results in better swimming performance. Experimental tests show that the turning radius increases with higher swing frequencies and lower stiffness, resulting in a larger turning radius. This experiment will help to improve the application of high-frequency wire-driven mechanisms in the study of robot fish movement and carry out more in-depth bionic research in the future.

## 1. Introduction

After millions of years of evolution, fish have acquired excellent swimming abilities in water, which has aroused great interest among scholars of bionics and oceanography. They have tried to incorporate the fish’s superb swimming skills into robotic fish. With continuous advancements in science and technology, research on robotic fish has matured. Researchers at the Massachusetts Institute of Technology in the United States successfully developed the world’s first bionic robotic tuna (Robo Tuna) in 1994 after observing the swimming of tuna for a long time [1]. As motors are widely used in robotic fish, Liang et al. [2] developed a tail fin propeller driven by a servo motor to verify the high maneuverability and reliability of fishtail fin propulsion. With the maturity of bionic technology, more and more robotic fish have been designed and developed to pursue a swimming posture closer to that of real fish. Research on robotic fish is no longer limited to the multi-link design driven by motors and propellers [3,4]. Although this design is effective, its mechanism is complex and inefficient, and the rigid connection between the joints leads to a low fit between the fish body swing and the swimming of real fish. In order to be more similar to real fish, Zhong proposed a robotic fish based on a wire drive mechanism [5,6]. The wire-driven mechanism can not only improve the swimming of the robotic fish and the swimming of real fish but also reduce the difficulty and complexity of control. However, due to its structural reasons, this traditional wire-driven robotic fish makes it difficult to achieve high-frequency swimming.

Although the movement of robotic fish based on wire-driven mechanisms is very similar to that of real fish, most studies have only explored its movement at low frequencies, which usually results in insufficient propulsion in the water. High-frequency movement can better simulate the rapid swimming and complex movements of fish and improve the swimming efficiency of robotic fish. Zhang et al. [7] designed a robotic fish with high-frequency vibration characteristics based on the characteristics of electromagnetically driven machinery and tuna imitation. The results showed that increasing the tail-flicking frequency can improve the swimming speed of the robotic fish to a certain extent. However, electromagnetic drive is not only difficult to control but also consumes a significant amount of energy. Yu et al. [8] developed a two-joint bionic robotic fish driven by a single motor. It is driven at high frequency by an eccentric wheel structure, with a swing frequency of more than 9 Hz and a maximum swimming speed of 3.07 BL/s. However, its flexibility is poorer than that of multi-joint robotic fish. Lauder’s team [9] continued to optimize the platform to achieve three different numbers of joint configurations. The experiment compared robotic fish with two, three, and four fish joints and analyzed their swimming performance. The results showed that increasing the number of joints in the fish’s body can improve swimming performance, but this will also increase the difficulty of control and reduce its flexibility. Although there have been many studies on the high-frequency swimming of robotic fish, most of them are multi-link or single-link mechanisms, and there are few studies on high-frequency wire-driven robotic fish, so we explored this field.

Liao [10] proposed a robotic fish equipped with a wire-driven dual-elastic tail with energy storage and passive flexibility, capable of achieving high-frequency swings of 12 Hz, thereby improving swimming performance. To further advance the research on high-frequency wire-driven robotic fish, Liao et al. [11] also designed an elastic robotic fish based on wire-driven technology, where the tail is actuated by a wire system controlled by dual servomotors to simulate natural fish movements. Studies have shown that at high frequencies, the amplitude has a more significant effect on improving swimming speed. However, the design of the elastic fishtail makes the drive structure more complicated, and frequent high-frequency swings easily lead to structural deformation. In addition, in 2022, S.C. van den Berg et al. [12] proposed a high-speed swimming bionic robotic fish OpenFish based on a cable mechanism. The system drives the cable through the continuous rotation of the DC motor, driving the coordinated movement of the flexible tail on both sides and improving propulsion efficiency during high-speed swimming. However, due to the limitations of flexible tail structure and cable material, OpenFish still faces challenges in achieving high frequency and high precision control, especially in complex water flow environments, and its control response is relatively limited.

The experimental results in this paper show that increasing the frequency can increase the swimming speed of the robotic fish to a certain extent, but too high a frequency will reduce the speed of the robotic fish [13]. The robotic fish with the lowest tail stiffness at 1 Hz has the best swimming performance. As the frequency increases to 4 Hz, the swimming speed of the fully rigid fishtail increases to the fastest. The turning performance of the robotic fish is better at low frequencies, and the stronger the stiffness of the robotic fishtail, the smaller the turning radius.

## 2. The Design and Modeling of the Robotic Fish

Inspired by biology, most bionic robotic fish imitate the design and swimming style of real fish to improve propulsion efficiency and flexibility. However, most current research on bionic robotic fish cannot take into account both bionic effects and high-frequency oscillations. Few people study the high-frequency motion of robotic fish while ensuring the same flexibility as real fish. Although there is still a long way to go to achieve the high-frequency oscillation movement posture of real fish, this paper has achieved the combination of the two to a certain extent by studying the high-frequency movement of tuna and proposing novel contents.

### 2.1. Mechanical Design of the Robotic Fish

**Figure 1:**
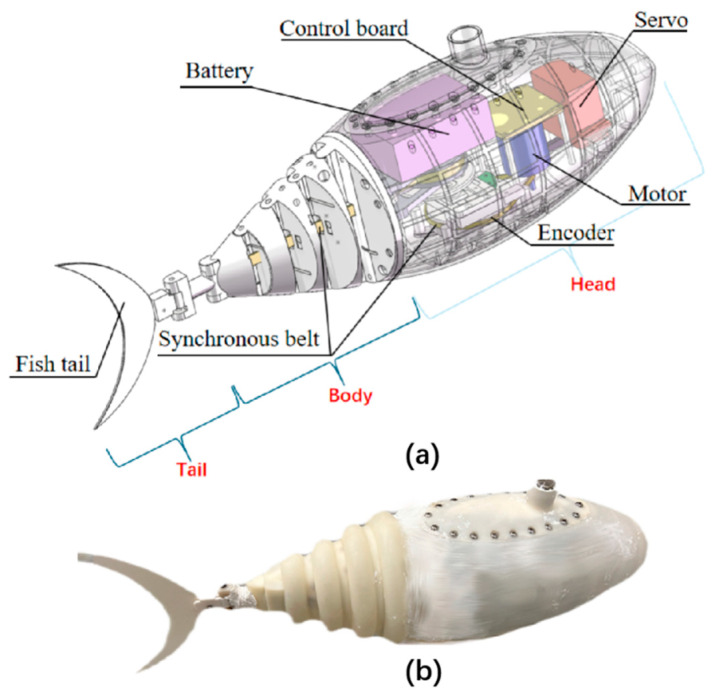
Overview and actual image of the robotic fish: (**a**) the overview design of the robotic fish; (**b**) robotic fish physical model.

The robotic fish designed in this paper adopts the (Body and/or Caudal Fin propulsion) BCF model of the tuna family. Figure 1a shows a robotic fish designed in imitation of tuna [14]. It consists of three parts: head, body, and tail. The head length is 201 mm, the body length is 129 mm, the tail length is 100 mm, and the total weight is 1610 g, as shown in Table 1. The head was manufactured using 3D printing technology and houses the control panel, servo, motor, battery, encoder, synchronous pulley, and transmission mechanism. Counterweights were added inside to balance gravity and buoyancy. The brushless motor was used as the power source, and the rotation of the motor was output to the synchronous pulley through a double sine mechanism. The synchronous pulley drives the synchronous belt to move, thereby realizing the swing of the fishtail. The body contains an active wire-driven mechanism with four joints and synchronous belts. One end of the synchronous belt is connected to the pulley, while the other end is fixed to the last joint of the body. When the synchronous pulley rotates, the synchronous belt is rotated one circle, the other side is extended, and the tail fin on the seabed is applied to collect. The fishtail is connected to the body through the tail joint. The tail connection joint can be equipped with torsion springs of different wire diameters to change the tail stiffness. Figure 1b is the visual model.

**Figure 2:**
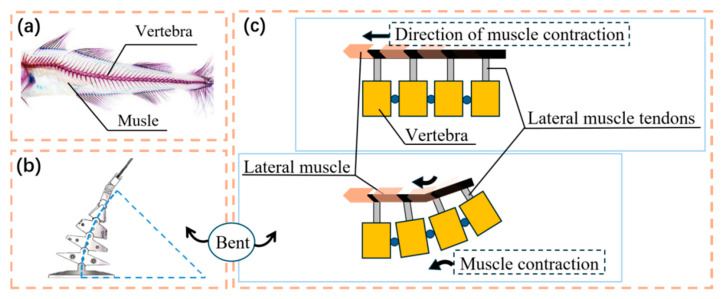
Overview of the skeleton structure and muscle arrangement of fish: (**a**) skeleton and muscle arrangement; (**b**) schematic diagram of the bending of the robotic fish’s body swing; (**c**) the spine bends due to muscle contraction.

In order to be closer to the swimming posture of real fish, we estimate the oscillating swimming of fish as a circular arc segment, as shown in Figure 2b. From the driving perspective, the curvature of the body arc segment was obtained by limiting the stolen. Figure 2a shows the fracture and resection arrangement of bony fish. The joints are used to simulate the vertebrae, and the axis is rotated at the connecting joints to constrain the joint movement [15]. Since the resection can only bear tension, the complex bending of the fish body on both sides requires the joint action of the sculpture: lateral contraction, as shown in the left side of the figure, the fish body bends, and vice versa. The structure of the fish and its body drive mechanism are very similar to the bionic wire drive mechanism, which can easily construct the body curve. As shown in Figure 2c, a synchronous belt is used to replace the resection of the fish. The two synchronous belts are stuck by the bearing and the toothed support wheel when passing through the vertebrae, ensuring that the joints can rotate synchronously without skipping teeth. At the same time, it also ensures that the synchronous belt on the steering wheel does not slip when the fish body swings at high speed, effectively increasing the stability of the robotic fish body when moving. In addition, the fish body is waterproofed by wrapping the soft rubber material fish skin prepared by 3D printing technology on the outside of the fish body [16]. The fish skin is fixed to the base of the robotic fish head and the last joint of the fish body with silicone rubber.

**Figure 3:**
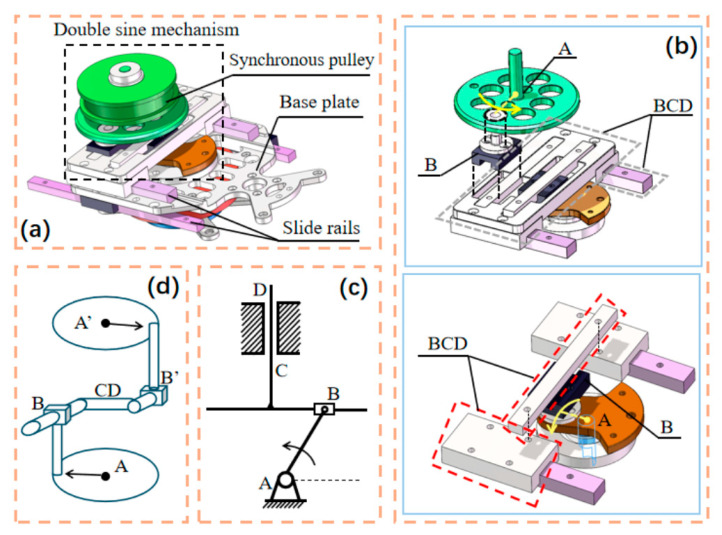
Double sine mechanism structure and schematic diagram: (**a**) overall schematic diagram of transmission mechanism (**b**) physical picture of double sine mechanism; (**c**) sine mechanism schematic diagram; (**d**) double sine mechanism schematic diagram.

Compared to traditional multi-link robotic fish driven by motors, wire-driven robotic fish better mimics the swimming posture of real fish. However, due to mechanical strength limitations and swing stability concerns, most research has focused on low-frequency motion. To enable high-frequency swinging, this paper designs a transmission mechanism based on a double sine mechanism. As shown in Figure 3a, the transmission mechanism consists of a fixed base, a linear guide rail, a dual-sine mechanism, and a synchronous wheel. Two linear slide rails are installed under the head of the robotic fish. The fixed base is connected to the slide rails, and the fixed base can be moved forward and backward by rotating the servo. Figure 3b is a structural diagram of the dual-sine mechanism. In principle, the dual-sine mechanism is composed of two oppositely placed sine mechanisms. By swapping the input and output of the two and then combining them, it can be ensured that the input of the motor can be output to the synchronous pulley according to the transmission ratio. Figure 3c shows a sine mechanism, which consists of a crank AB, a slider B, and vertically intersecting slides BCD. When crank AB rotates, slider B makes a circular motion. Since the vertically intersecting slides offset the horizontal motion, the motion trajectory output from the C end changes with time as a sine change, that is, a sine motion in the vertical direction [17]. By using the output of a sine mechanism as the input of another sine mechanism, a double sine mechanism can be formed. The schematic diagram of the double sine mechanism is shown in Figure 3d. The motor signal is input from the A end, driving the sine wheel to rotate. The slide CD perpendicular to the slider B can only reciprocate in one direction under the restriction of the limit device. At the same time, the slide rail where slider B’ is located is also perpendicular to CD and parallel to the slide rail where slider B is located. Due to the different transmission ratios, slider B’ is driven by CD to reciprocate in an incomplete circle at an initially determined middle position, thereby outputting a rotation signal to the A’ end. The synchronous pulley is fixedly connected to A’, so when the motor signal is input, the synchronous pulley also reciprocates.

Although the output of a single sine structure can ensure that the speed is symmetrical compared to the center point, the lateral deflection force received by the synchronous pulley is always applied to a single side of the limit device. When the movement is long-term or high-frequency, the output end of the sine mechanism will be deformed due to excessive lateral force during the movement and additional vibration caused by the body, resulting in asymmetric output of the sine mechanism, thereby affecting the fishtail swing. The dual sine mechanism is designed to solve this problem. Taking Figure 3d as an example, when slider B moves along the slide rail, the output end is connected to the other slide rail. At this time, the force applied to the limit device is dispersed by the movement of the slider B’, reducing the local force and making the output movement more stable.

As can be seen from the body part of Figure 1, the synchronous belt replaces the traditional steel rope as the “muscle” of the robotic fish, which is used to drive the body to swing. The output end of the double sine mechanism is connected to the synchronous pulley in Figure 3a, and the synchronous belt is fixed on the synchronous pulley. The body part of the robotic fish contains four joints. There are holes on both sides of each joint to pass the synchronous belt. Synchronous gears are installed inside the joint holes to engage the synchronous belt. This design ensures that the robotic fish can swing at high frequencies without breaking the line or slipping the teeth. The experimental test results show that the structure can withstand a maximum of 24 Hz swing while the transmission mechanism is still stable, and the synchronization does not loosen or break. In contrast, the robotic fish driven by steel rope will break when the swing frequency exceeds 12 Hz. When the sine mechanism is used as the transmission mechanism, the synchronous wheel will loosen when the swing frequency exceeds 15 Hz, resulting in a decrease in the swing angle of the robotic fish. The experimental results show that the double sine mechanism can increase the stability of the robotic fish during high-frequency movement while reducing the wear of the internal transmission mechanism under high-frequency movement [18].

### 2.2. Electronics and Control System

**Figure 4:**
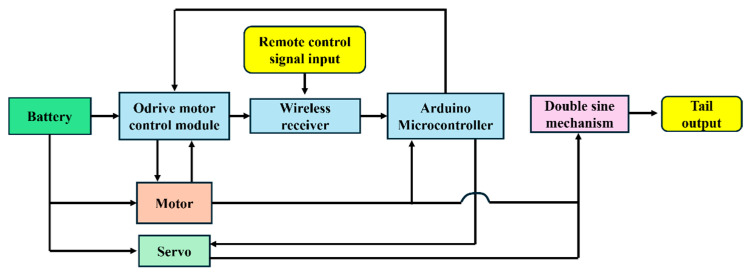
Block diagram of electromechanical control system.

All electronic devices are installed in the head of the robotic fish. The power source is a 22.2 V 6S lithium battery (850 mAh), which powers the motor control module, microcontroller, and wireless module via a step-down converter. The system uses a 5 V Savox SW-1210SG brushless motor, while the auxiliary steering system is powered by a KST X20-7512 servo. The sensor system is composed of an AS5047P encoder, which can control the motor speed according to the feedback signal to achieve speed control or turning movement of the robotic fish. The Arduino Seeed Studio XIAO SAMD21 serves as the central computer, receiving signals from the wireless module, processing sensor data, and sending control commands to the motor and servo. When the robotic fish is turned on, the servo drives the fixed bottom plate back to the calibration start position, and the remote control handle can send a frequency signal. When the wireless module receives the corresponding remote control signal, the motor will rotate according to the frequency, and the robotic fishtail swings its body at this frequency to swim straight. The servo signal and the motor signal do not interfere with each other. This is because the turning signal is only sent to the steering auxiliary servo, which is used to adjust the swimming direction. When the external device sends the servo signal, the steering auxiliary servo drives the bottom plate to move a certain distance, causing the middle position of the robotic fish to deflect. Figure 4 is the control system framework.

## 3. Modeling and Design of Robotic Fish

### 3.1. Kinematic Model of Robotic Fish Swimming

**Figure 5:**
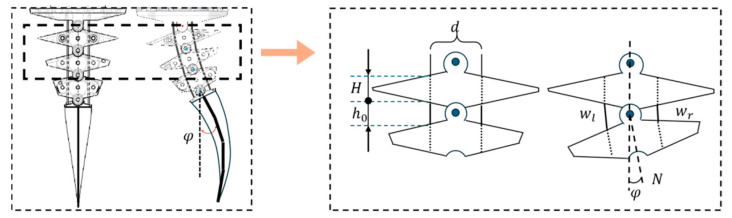
Schematic diagram of the robotic fish’s body swing.

Figure 5 is a cross-sectional view of adjacent joints when the robotic fish is swimming, and its body is bent. In this figure, d is the vertical distance between the synchronous belts, H is the thickness of the joints, $h_{0}$ is the distance between the joints in the initial state, φ is the angle of the fish body relative to the central axis, N is the number of joints of the fish body, and $w_{l}$ and $w_{r}$ are the left and right distances between adjacent joint holes after the fish body swings [19,20]. Because of the existence of the joint torsion spring, each joint can rotate evenly to the same side under the drive of the synchronous belt, so it can be assumed that the rotation angle of each joint is the same. φ/N is the rotation angle of each joint. From this, the length change in the two synchronous belts can be calculated as follows:

(1) $${∆w}_{l}=d\sin\frac{\varphi }{2N}-2h_{0}\sin^{2}\frac{\varphi }{4N}$$

(2) $${∆w}_{r}=-d\sin\frac{\varphi }{2N}-2h_{0}\sin^{2}\frac{\varphi }{4N}$$

where ${∆w}_{l}$ and ${∆w}_{r}$ are the variable lengths of the left and right synchronous belts, respectively. Since the rotation angle φ/N between each joint is very small and can be almost ignored, the binomial in the above formula can be omitted, and the entire expression can be simplified to the following:

(3) $${∆w}_{l}=d\sin\frac{\varphi }{2N}$$

(4) $${∆w}_{r}=-d\sin\frac{\varphi }{2N}$$

When the upper synchronous pulley rotates by an angle of α driven by the double sine mechanism, the length ∆l of the synchronous belt rotating around the rotating axis of the upper synchronous pulley is as follows:

(5) $$∆l=2\pi l\frac{\alpha }{360^{{}^{\circ}}}$$

In Formula (5), l is the radius of the upper synchronous belt pulley. Driven by the rotation of the upper synchronous belt pulley, the length of the synchronous belt on the right side of the fish body becomes shorter, while the length of the synchronous belt on the left side becomes longer. From (3) and (4), it can be seen that the shortened length of the synchronous belt on the right side is equal to the increased length of the synchronous belt on the left side. Because the synchronous belt passes through the idler wheel and meshes, it can be determined that the synchronous belt does not deform under the action of tension; that is, the total length does not change. Therefore, the following formula is obtained:

(6) $$∆l={N|∆w}_{l}\left|=N\right|{∆w}_{r}|$$

Substituting Formulas (3) and (5) into Formula (6), we can obtain the following:

(7) $$\varphi =2N\sin^{-1}\frac{\pi l\alpha }{180^{{}^{\circ}}Nd}$$

where *φ* is the swing angle of the fish body, α is the rotation angle of the synchronous pulley. Since the inverse sine function in (7) is small, it can be simplified to the following:

(8) $$\varphi =\frac{2l\alpha }{d}$$

Since the motor output angle is β, r: R is the ratio of the input axis offset to the output axis offset of the double sine mechanism, and the input gear ratio of the motor and the double sine mechanism is m : M, the synchronous belt rotation length ∆L of the sine mechanism input can be obtained:

(9) $$∆L=\sin\frac{m\beta }{M}$$

According to the transmission characteristics of the double sine mechanism, the rotation angle of the upper synchronous pulley can be obtained as follows:

(10) $$\alpha =\sin^{-1}\left(\frac{r}{R}\sin\frac{m\beta }{M}\right)$$

Substituting Equation (10) into Equation (8), the relationship between the motor rotation angle and the machine tail swing angle can be obtained after simplification:

(11) $$\varphi ={\frac{2l}{d}\sin}^{-1}\left(\frac{r}{R}\sin\frac{m\beta }{M}\right)$$

Table 2 provides the transmission ratio between the synchronous pulley and the double sine mechanism. Substituting these data into Equation (11), when the β angle reaches its maximum, we can calculate that the maximum swing angle φ of the robotic fish is 45°.

When the robotic fish turns, the steering gear provides a transfer deflection angle to drive the base plate of the double sine mechanism to move, and the moving distance is x (x is the absolute value of the distance from the calibration starting position of the base plate. When the base plate moves forward relative to the starting position, the robotic fish turns left; when the base plate moves backward relative to the starting position, the robotic fish turns right.) At this time, the tail swing angle of the robotic fish can be rewritten as follows:

(12) $$\varphi ={\frac{2l}{d}\sin}^{-1}\left(\frac{r}{R}\sin\frac{m\beta }{M}+x\right)$$

When the robotic fish receives a turning signal, the servo drives the bottom plate of the double sine mechanism to move, thereby providing a deflection angle. The new center position has an additional angle compared to the original fish body. The sensor feedback system obtains the current swing direction and position of the fishtail and switches the speed value at the maximum left and right swing position of the fishtail. The speeds generated by the left and right motors when working alternately are different, so a speed difference is generated, thereby providing the deflection force required for steering. The robotic fish in this article achieves differential turning based on this.

### 3.2. Hydrodynamics Modeling of Robotic Fish

To study the motion performance of the robotic fish, we modeled its movement in two modes: straight swimming and turning. The modeling during the swimming process includes two parts: the head and the tail. The fish’s head is modeled as a rigid body, with hydrodynamic forces and thrust from the tail treated as external forces. The dynamic model of the horizontal plane is established according to the Newton–Euler equation. We model the tail swing using a pseudo-rigid body approach to simulate the deformation during movement. The Morison [21] equation is then applied to calculate the hydrodynamic forces generated by the tail’s motion.

**Figure 6:**
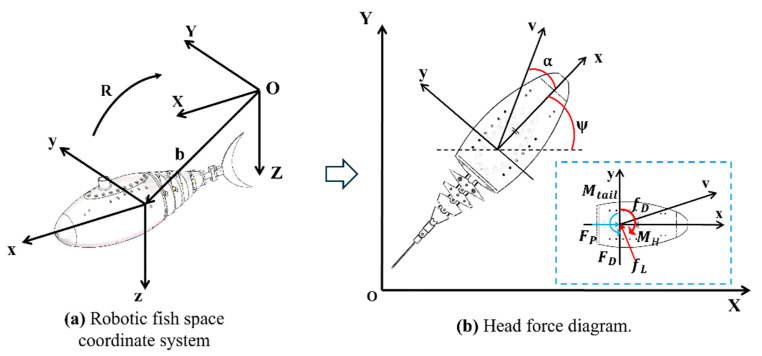
Robotic fish space motion coordinates.

In order to facilitate the analysis of the movement of the robotic fish underwater, a spatial motion coordinate system is established, as shown in Figure 6. The coordinate system includes the inertial coordinate system OXYZ and the body coordinate system oxyz. Assuming that the center of gravity of the head coincides with point c,$v={\left(v_{1},v_{2},v_{3}\right)}^{T}$ and $\Omega ={\left(\Omega_{1},\Omega_{2},\Omega_{3}\right)}^{T}$ represent the speed and angular velocity of the robotic fish in the body coordinate system, respectively. $v_{1},v_{2},v_{3}$ are the velocity components along the x, y, and z axes respectively; $\Omega_{1},\Omega_{2},\Omega_{3}$ are the angular velocity components of the x, y, and z axes, respectively. The transformation relationship between the body coordinate system and the inertial coordinate system is expressed by the Euler angle, which includes the pitch angle θ, the yaw angle ψ, and the roll angle ϕ. The pitch angle θ is defined as the angle between the ox axis and the OXY plane, which is positive when the ox axis is facing upward. The yaw angle ψ is defined as the angle between the projection of the ox axis on the OXY plane and the OX axis, which is positive when the projection line is between the OX axis and the OY axis. The roll angle ϕ is defined as the angle between the oxz plane and the longitudinal plane where the ox axis is located. It rotates around the ox axis according to the right-hand rule. When the finger passes through the longitudinal plane first, ϕ is positive. α is the angle of attack of the v velocity vector relative to the x-axis.

The positional relationship between the body coordinate system and the inertial coordinate system is represented by the vector b, and R is the rotation matrix from the body coordinate system to the inertial coordinate system. The modeling of the gliding robot fish in this paper mainly focuses on its movement on the horizontal plane OXY without considering the floating and diving movements. The coordinate relationship of the OXY plane is shown in Figure 6b.

When the robotic fish is in water, its head can be regarded as a rigid body. The motion of a rigid body in water can be expressed by the Kirchoff equation, so there is the following formula:

(13) $$\dot{P}=P\times \Omega +F_{e}$$

(14) $$\dot{H}=H\times \Omega +P\times v+M_{e}$$

where P is the total momentum of the robotic fish head, H is the total angular momentum of the head, and the external force and torque applied to the center of gravity of the head are $F_{e}={\left[F_{x},F_{y},F_{z}\right]}^{T}$ and $M_{e}={\left[M_{x},M_{y},M_{z}\right]}^{T}$, respectively.

Since we are focusing on the motion of the plane and not on the vertical motion of the robotic fish, we do not consider the effect of the change in the center of gravity of the tail on the center of gravity of the head during the swinging process. The robotic fish is affected by the combined forces from fluid interaction and tail fin motion. Therefore, this paper also needs to consider the effects of the drag $f_{D}$, lift $f_{L}$, hydrodynamic torque $M_{H}$, water flow impact force $F_{D}$, thrust $F_{p}$, and thrust torque $M_{tail}$, generated by the tail fin swing. In summary, the swimming dynamics equation of the robotic fish can be simplified as follows:

(15) $$m_{h}\dot{v_{1}}=m_{h}v_{2}\Omega_{3}+F_{x}$$

(16) $$m_{h}\dot{v_{2}}=-m_{h}v_{1}\Omega_{3}+F_{y}$$

(17) $$J_{3}\dot{\Omega_{3}}=M_{tail}+M_{H}$$

where $m_{h}$ is the weight of the robotic fish head, and $J_{3}$ is the tensile inertia of the z-axis at the center of gravity of the robotic fish. At the same time, the external force and torque can be expressed by the content given in Figure 6.

(18) $$F_{x}=F_{P}+f_{L}\sin\alpha -f_{D}\cos\alpha$$

(19) $$F_{y}=F_{D}-f_{L}\cos\alpha -f_{D}\sin\alpha$$

The calculation formula of the hydrodynamic force on the head of the robotic fish during movement is as follows:

(20) $$f_{L}=\frac{1}{2}\rho_{w}C_{HL}A_{H}v^{2}\alpha_{L}$$

(21) $$f_{D}=\frac{1}{2}\rho_{w}C_{HD}A_{H}v^{2}$$

(22) $$M_{H}=-C_{HM}{\Omega_{3}}^{2}sgn(\Omega_{3})$$

Among them, $C_{HL}$, $C_{HD}$ and $C_{HM}$ are the lift coefficient, drag coefficient, and torque coefficient of the robotic fish head, respectively; $\alpha_{L}$ represents the average angle of attack of the fish’s body during swimming and swinging; $A_{H}$ is the surface area of the robotic fish head; and $\rho_{w}$ is the water density.

**Figure 7:**
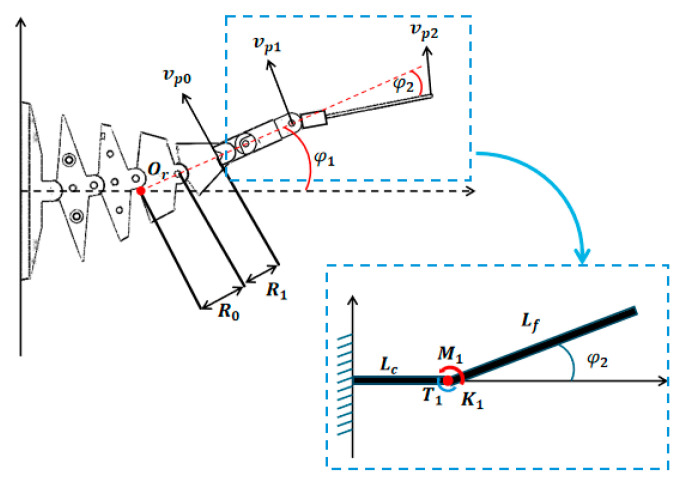
Tail kinematics model.

Figure 7 is the tail kinematic model of the robot fish.The thrust of the robotic fish’s forward movement and the torque when turning are both generated by the tail swing. The force analysis of the tail swing is crucial to the kinematic mechanics modeling of the robotic fish. The model is shown in Figure 6. This paper adopts the 1R tail rigid body model, which consists of two rigid rods connected by a hinge and a torsion spring. To simplify the calculation, $L_{c}$ is the length of the active tail section, $L_{f}$ is the length of the passive tail section, $K_{1}$ is the torsion spring constant, $T_{1}$ is the torque generated by the torsion spring, $M_{1}$ is the hydrodynamic torque that causes the tail to bend, and $\varphi_{2}$ is the deflection angle of the second tail section.

The torque $T_{1}$ generated when the torsion spring deflects $\varphi_{2}$ can be approximately calculated as follows:

(23) $$T_{1}=K_{1}\varphi_{2}$$

Under the combined effect of the hydrodynamic torque and the torsion spring, the deflection acceleration of the second tail section is as follows:

(24) $$\ddot{\varphi_{2}}=\frac{\left(T_{1}+M_{1}\right)}{J_{c}}$$

where $J_{c}$ is the moment of inertia of the second tail model, and the torsion spring constant can be obtained by calculating the following equation:

(25) $$K_{1}=\frac{Ed^{4}}{10.8DN}$$

where E is the elastic modulus (200 GPa for steel), d is the wire diameter, D is the average diameter, and N is the number of turns.

In the process of driving the wire to drive the joint to swing, in order to obtain the swing speed of the end joint, the center line of the end joint can be extended to intersect with the x-axis of the fuselage. The intersection $O_{r}$ is used as the rotation center of the end joint; $\varphi_{1}$ is the deflection angle of the end join; $v_{p0},v_{p1},v_{p2}$ correspond to the speed of $P_{0},P_{1},P_{2}$ points, respectively, and $R_{0},R_{1}$ are parameters for calculating the rotation radius, where $R_{1}$ is a constant, and $R_{0}$ needs to be calculated based on the current deflection angle $\varphi_{1}$. According to the geometric relationship, the following can be calculated:

(26) $$R_{0}=\left\{\begin{array}{c} \;\;\;\frac{H_{0}\left(\sin\frac{\varphi_{1}}{4}+\sin\frac{2\varphi_{1}}{4}+\sin\frac{3\varphi_{1}}{4}\right)}{\sin\varphi_{1}}\;\;\;\;\;\;\;\;\;\;\;\;\;\;\;\;\;\varphi_{1}\neq 0\\ \;\;\;\;\;\;\;\;3H_{0}\;\;\;\;\;\;\;\;\;\;\;\;\;\;\;\;\;\;\;\;\;\;\;\;\;\;\;\;\;\;\;\;\;\;\;\;\;\;\;\;\;\;\;\;\;\;\;\;\;\;\;\;\;\;\;\;\;\;\;\;\varphi_{1}=0 \end{array}\right.$$

The speed of any point between the angular velocities $\omega_{t}=\frac{d\varphi_{1}}{dt}=\dot{\varphi_{1}}$, $P_{0}$, and $P_{1}$ of the tail swing can be expressed as follows:

(27) $$V_{P1}=\omega_{t}\times r_{pm}$$

where $r_{pl}$ is the position vector between $O_{r}$ and point $P_{1}$, the velocity magnitude at $P_{1}$ is $V_{P1}=\dot{\varphi_{1}}(R_{0}+R_{1}+l)$, and l is the distance between $P_{0}$ and $P_{1}$. According to the velocity relationship between the rods, the velocity of any point $P_{m}$ between $P_{1}$ and the end of the tail fin consists of two parts: the velocity at point $P_{1}$ and the linear velocity around point $P_{1}$, which can be expressed as follows:

(28) $$V_{pm}=V_{P1}+\omega_{c}\times r_{pm}$$

where $\omega_{c}$ is the bending angular velocity of the hinge of the pseudo-rigid body model, its magnitude is $\omega_{c}=\dot{\varphi_{2}}$, and $r_{pm}$ is the position vector from point $P_{1}$ to $P_{m}$. According to the Morison equation, the hydrodynamic forces on the flexible middle section and the tail fin during swinging mainly include drag force and inertia force, where the drag force is the force on the tail caused by the velocity of the water flow particle and the inertia force is the force on the tail caused by the acceleration of the water flow particle. The hydrodynamic force on any point n of the flexible middle section and the tail fin can be expressed as follows:

(29) $$f(n)=-\frac{1}{2}\rho_{w}C_{d}V_{n}\left|V_{n}\right|d(n)-\frac{1}{4}\pi d^{2}(n)\rho_{w}C_{m}{\dot{V}}_{n}$$

where $C_{d}$ and $C_{m}$ are the drag coefficient and inertia coefficient, respectively, $V_{n}$ is the velocity at point n, and d(n) is the chord length of the section at point n of the tail. The hydrodynamic force on the entire tail can be obtained by calculating the sum of the hydrodynamic forces from $P_{0}$ to $P_{1}$ and from $P_{1}$ to $P_{2}$.

(30) $$F_{tail}=\left(\begin{array}{c} F_{P}\\ F_{D} \end{array}\right)=\int_{0}^{L_{c}+L_{f}}f(x)dx$$

The torque $M_{tail}$ generated by the tail swing is as follows:

(31) $$M_{tail}=\int_{0}^{L_{c}+L_{f}}r_{x}\times f(x)dx$$

where $r_{x}$ is the position vector from the coordinate origin o of the robotic fish body to the tail action point.

## 4. Simulation and Experimental Research

**Figure 8:**
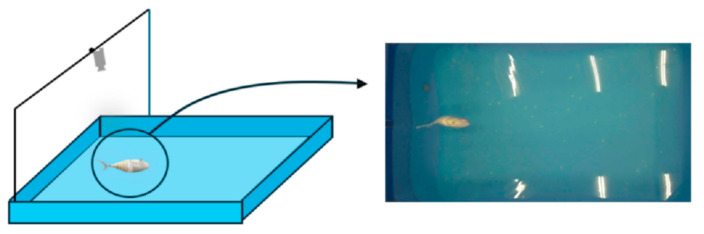
Robotic fish swimming experimental platform.

In order to verify the swimming performance of the transmission structure designed in this paper on the robotic fish, a series of underwater swimming experiments at different frequencies were conducted on robotic fish with different tail stiffness. The tail connection joint can replace the torsion spring to change the tail stiffness of the robotic fish. This experiment was equipped with 0.8 mm torsion spring wire diameter, 1.0 mm torsion spring wire diameter, and full rigid fishtail (hereinafter collectively referred to as 0.8 mm stiffness fishtail, 1.0 mm stiffness fishtail, and full rigidity fishtail). The whole experiment was mainly divided into two parts: a straight swimming experiment and a turning swimming experiment. In addition, the swimming experiment was also to further verify the accuracy of the established mathematical model. The size of the pool for the experiment was 2 m × 3 m, and the depth was 40 cm. The experiment was carried out at room temperature of 25 °C. A camera was installed above the pool, as shown in Figure 8, to upload the picture of the robotic fish swimming to the computer monitoring interface. This paper uses MATLAB as the simulation platform to implement the dynamic model simulation, with the environment defined as a flow environment. In conjunction with the dynamic modeling in Chapter 3.2, the simulation fully accounts for the dynamic effects of water flow on the robot fish’s head and tail fins.

Both the forward swimming and turning experiments assessed the swimming performance of three groups of robotic fish with different tail stiffness at various frequencies [22]. The forward swimming experiment measured the swimming speed of the robotic fish with different tail stiffnesses at frequencies ranging from 1 to 7 Hz, while the turning experiment measured the turning radius of the robotic fish at frequencies ranging from 1 to 3 Hz.

### 4.1. Forward Swimming

**Figure 9:**
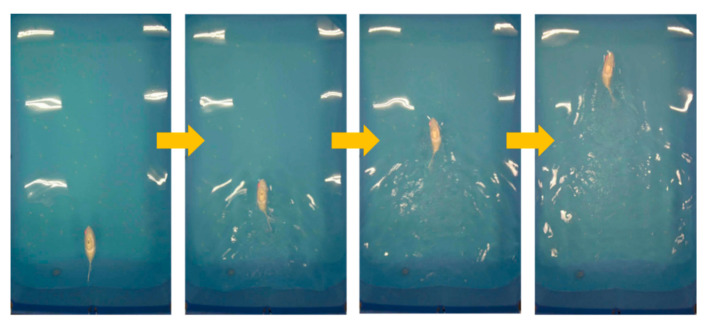
Forward swimming process.

**Figure 10:**
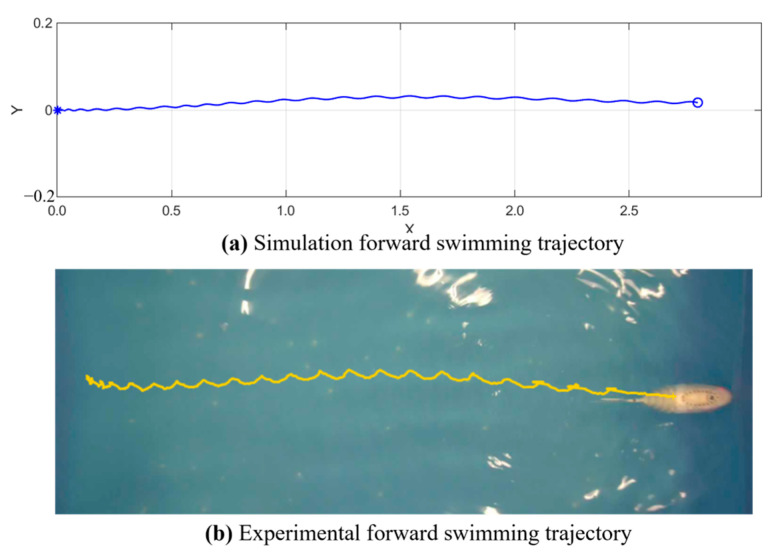
Simulation and experimental motion trajectory.

The linear swimming part mainly explores the effect of different tail stiffness and frequency on the swimming speed of the robotic fish. In order to refine the experimental process, the experiment tested three groups of robotic fish with different tail stiffness in the range of 1–7 Hz. Three experiments were conducted at each frequency [20], and the average of the three experimental results was taken as the experimental result, with a total of 21 groups of experimental results. Figure 9 is a process diagram of the robotic fish swimming in a straight line. When the different parameters were tested experimentally, the 21 different parameter groups were simulated at the same time. The simulation result and actual trajectory of one linear swimming are shown in Figure 10.

**Figure 11:**
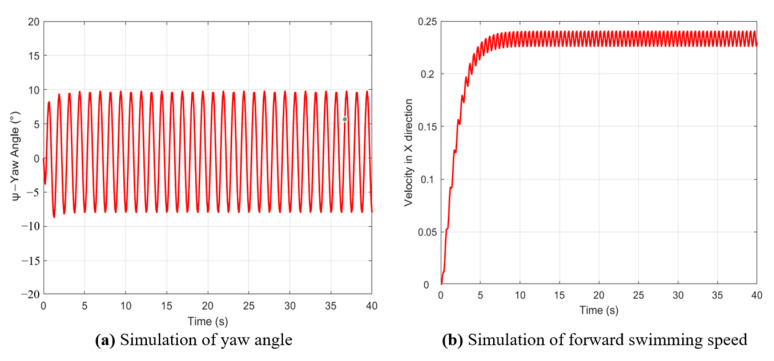
Simulation of yaw angle and forward swimming speed.

Figure 11a,b show the yaw angle and forward swimming speed obtained from the simulation, respectively. While the simulation of the yaw angle deviates from the actual yaw angle observed in the swimming experiment, this discrepancy arises because the robot fish’s tail does not remain perfectly centered when it starts swinging, leading to an asymmetric swing waveform. On the other hand, the simulation of swimming speed closely matches the experimental swimming speed for this group.

**Figure 12:**
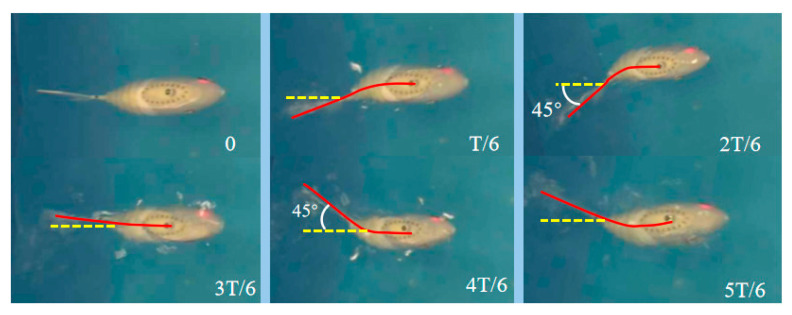
Image of a full rigid fishtail during a complete swing cycle.

From the comparison chart of simulation results and experiments, it can be found that the straight swimming trajectory of the robotic fish is consistent with the simulation results; the overall deviation of the yaw angle is not large, and it corresponds to the swimming trajectory. In addition, this experiment intercepted three groups of video frames of robotic fish with different tail stiffness flapping in the water to verify whether the maximum swing angle of the robotic fish swimming in a straight line is consistent with the kinematic model. In order to reduce the interference of water resistance during high-frequency swinging, taking the swing frequency of 2 Hz as an example, Figure 12 is an image of a full rigid fishtail in a complete swing cycle. Six frames of images were intercepted within T, namely 0, T/6, 2T/6, 3T/6, 4T/6, and 5T/6. In each frame of the image, the red solid line is the fish body swing curve, and the yellow dotted line is the median direction. The experimental results show that the maximum swing angle of straight swimming is about 45°, which is consistent with the result calculated by Formula (11). For the 0.8 mm stiffness fishtail and 1.0 mm stiffness fishtail, due to the influence of water resistance on both sides, when the active section body bends, the torsion spring cannot generate sufficient restoring force in time, resulting in the fishtail swing angle being less than 45°, but the bending angle of the joint at the end of the active section body is still 45°.

**Figure 13:**
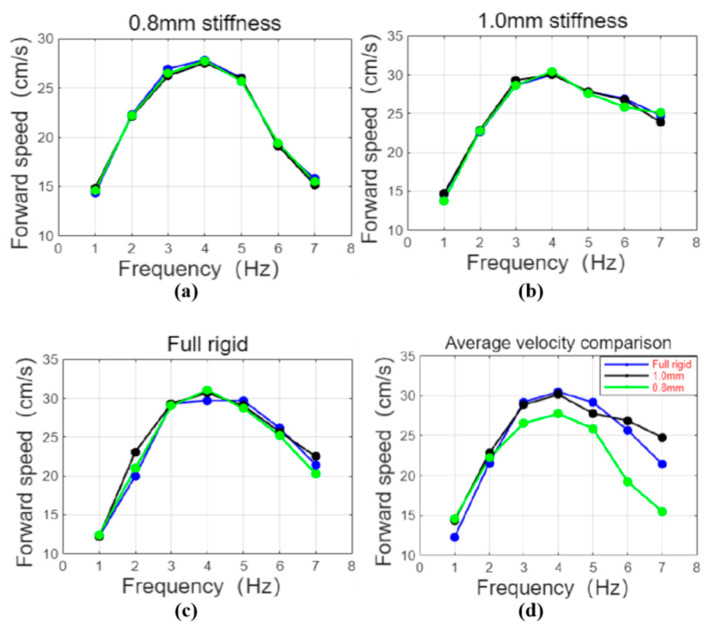
Results of the swimming speed of fishtails with different stiffness at different frequencies: (**a**–**c**) the experimental results of the forward swimming speed of the fishtail with 0.8 mm stiffness, 1.0 mm stiffness, and full stiffness at a frequency of 1–7 Hz; (**d**) comparison of average speeds of robotic fish with different tail stiffness.

In addition, the experimental measurement results of the swimming speed of three groups of fishtails with different stiffness at different frequencies are shown in Figure 13. The experimental results show that with the increase in frequency, the swimming speed of the three groups of robotic fish all tend to increase first and then increase. When the frequency reaches 4 Hz, the swimming speed begins to decrease. The reason is that the stiffness of the passive joint of the fishtail is relatively low. Before the swing frequency reaches 4 Hz, the swing amplitude of the tail fin is large, the propulsion efficiency is high, and the swimming speed is positively correlated with the frequency. When the swing frequency of the fishtail exceeds 4 Hz, the passive joint of the fishtail cannot respond in time, and a waveform will appear, resulting in a significant decrease in the swing amplitude of the tail fin. When the frequency continues to increase, the swing amplitude of the end of the fishtail continues to decrease, resulting in a negative correlation between the swimming speed and the frequency at this time. At the same time, when it reaches 7 Hz, the active joint of the robotic fish does not respond in time, and the swimming speed of the robotic fish drops rapidly at this time, so it is not within the scope of the study. Due to the existence of the active joint torsion spring, a higher frequency response can be achieved by replacing the joint torsion spring with a larger stiffness, but this is not discussed in this experiment.

Figure 13d is a comparison of the average values of the experimental results. When the swing frequency is 1 Hz, the swimming performance of the robotic fish using a 0.8 mm fishtail joint torsion spring is the best, with a speed of 0.34 times the body length, while the swimming performance of the robotic fish using a fully rigid fishtail joint is the worst, with a speed of only 0.285 times the body length. This is due to the existence of inertia and water resistance; the 0.8 mm torsion spring can undergo a large deformation, so the fishtail swing angle of the robotic fish is larger when it swings. Similarly, the swimming speed of the robotic fish using a 1.0 mm fishtail joint torsion spring is also slightly higher than that of the robotic fish using a fully rigid fishtail joint. However, as the swing frequency gradually increases to 3 Hz, the swimming performance of the robotic fish using a fully rigid fishtail joint gradually becomes better than the other two groups of rigid fishtails. This is because the passive section of the fishtail with too low stiffness will cause a slower response as the frequency increases, thereby affecting the swimming speed. When the frequency reaches 4 Hz, the swimming speed of the robotic fish reaches its maximum, which is 0.71 times the body length. When the frequency exceeds 4 Hz, the speed begins to decrease, and the swimming speed is negatively correlated with the increase in frequency. Since the 0.8 mm fishtail joint torsion spring is more easily deformed, the passive section of the fishtail cannot respond in time at high frequencies, and the swimming speed slows down rapidly. This is also the reason why the swimming speed of the robotic fish using this set of rigid fishtails in high-frequency mode lags far behind the other two sets of rigid fishtails. Although the overall swimming speed of the robotic fish slows down with the increase in frequency, when the frequency exceeds 5 Hz, the swimming speed of the robotic fish using a 1.0 mm fishtail joint torsion spring is better than that of the robotic fish using a fully rigid fishtail joint. Analysis shows that when the frequency is too high, the swing of the active section of the fishtail begins to fail to respond quickly, resulting in a large water resistance of the fully rigid fishtail, which leads to a large decrease in swimming speed. However, due to the characteristics of the torsion spring, the passive section of the fishtail of the robotic fish using the 1.0 mm fishtail joint torsion spring will still swing outward for a distance along the original swing direction under the action of inertia, making its swing angle larger, so the water resistance is relatively small, and the swimming speed decreases less. When the swing frequency is higher than 7 Hz, the swimming speed of the robotic fish continues to decrease and is unstable, so no more test records are made.

### 4.2. Turning Swimming

Turning is another important part of the robotic fish swimming mode. The robotic fish designed in this paper relies on the turning auxiliary servo to control the movement of the fixed bottom plate to change the middle position of the body swing. Specifically, the output of the double sine mechanism is offset by the movement of the bottom plate, resulting in a change in the standard sine motion, thereby changing the symmetry center of the sine motion. At the same time, the program controls the two sides of the robotic fish’s motion in the middle position to produce differential motion, and the two work together to complete the robotic fish’s turning. The accuracy of the swimming motion model is verified by comparing the experimental results with the swimming simulation results.

**Figure 14:**
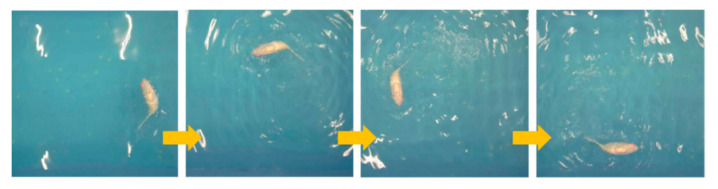
Process diagram of the robotic fish turning and swimming.

**Figure 15:**
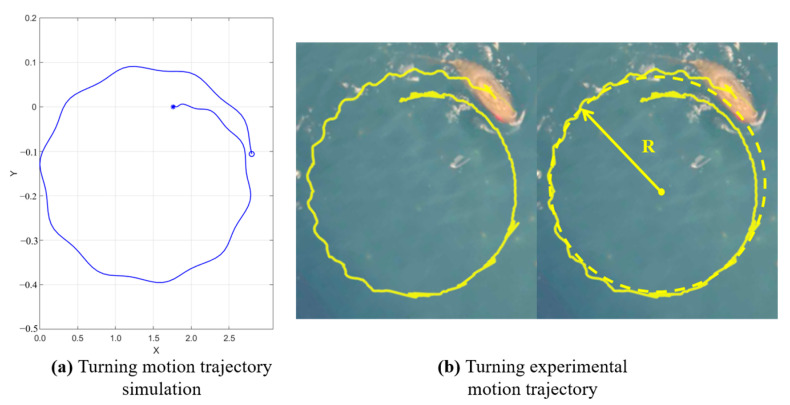
Turning experiment and simulation trajectory.

The experiment tested the turning of three groups of robotic fish with different tail stiffness in the range of 1–3 Hz. Three experiments were conducted at each frequency, and the average of the three experimental results was taken as the experimental result. The experimental results were taken at intervals of 0.5 Hz, and a total of 15 groups of experimental results were obtained. Figure 14 is a process diagram of the robotic fish turning and swimming. When the different parameters were tested experimentally, the 15 different parameter groups were simulated at the same time. The simulation results and actual trajectories of one-turning swimming are shown in Figure 15.

**Figure 16:**
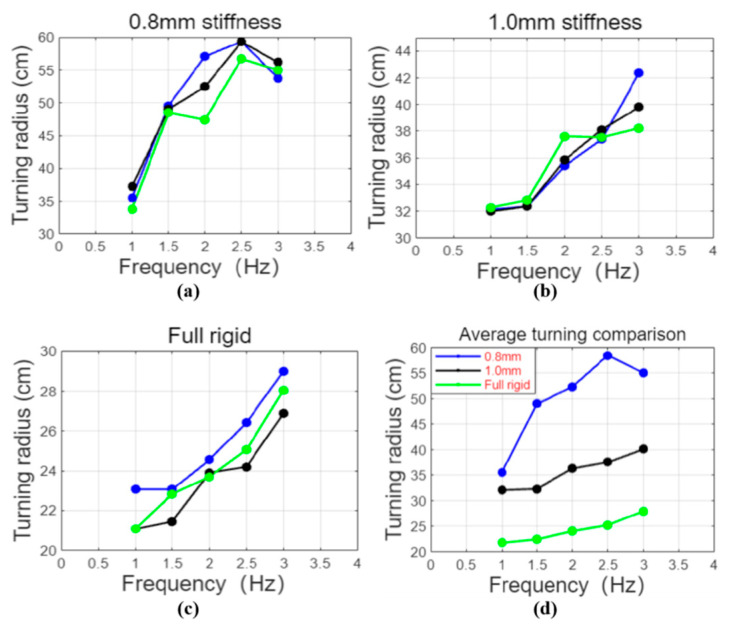
Results of turning radius of fishtail with different stiffness at different frequencies: (**a**–**c**) the experimental results of the turning radius of the fishtail with 0.8 mm stiffness, 1.0 mm stiffness, and full rigidity at 1–3 Hz frequency; (**d**) comparison of turning radius of robotic fish with different tail stiffness.

**Figure 17:**
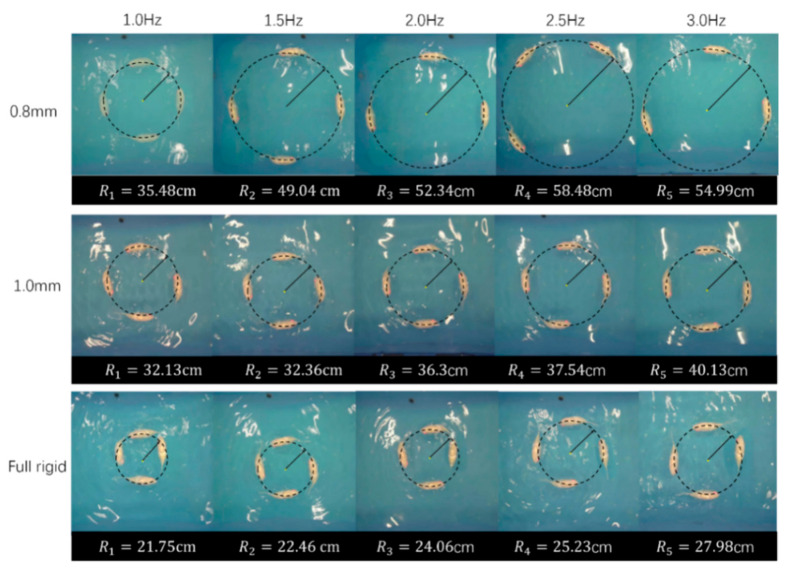
Turning results of fishtails with different stiffness at different frequency.

The simulation results are consistent with the actual experimental test results. Overall, the turning performance of the robotic fish designed in this paper is highly correlated with the results calculated by the dynamic model, which also proves the accuracy of the swimming model to a certain extent. Figure 16 is a schematic diagram of the robotic fish turning comparison under the line drive frequency of 1~3 Hz. It can be seen that the high-frequency line drive robotic fish designed in this paper can achieve a good turning effect. The three sets of experimental data all show that the turning radius increases with the increase in the robotic fish swing frequency. Among them, the robotic fish using a fully rigid fishtail joint has the smallest turning radius at a frequency of 1 Hz, which is 21.75 cm, about 0.51 body lengths. The robotic fish using a 0.8 mm fishtail joint torsion spring has the largest turning radius. This is because during differential steering, the lower the stiffness of the tail joint, the easier it is to sweep out a larger steering angle at a certain steering speed [23,24], thereby increasing the forward speed of the robotic fish and resulting in a larger swimming radius of the robotic fish [25,26,27]. High-frequency swinging increases the forward speed of the robotic fish, resulting in a larger turning radius. Therefore, the higher the swing frequency, the larger the turning radius; the lower the swing frequency, the smaller the turning radius. However, when the frequency exceeds 2.5 Hz, the turning radius of the robotic fish using a 0.8 mm fishtail joint torsion spring decreases. This phenomenon is caused by the low stiffness of the tail. When the frequency is too high, the passive section of the fishtail cannot respond in time, so the steering angle swept by the fishtail decreases, resulting in a decrease in the turning radius. At the same time, due to the presence of the variable central axis mechanism, the steering effect of the robotic fish is significantly improved compared to the traditional motor-driven differential steering robotic fish, but there is still a gap compared to the traditional servo-driven robotic fish. Figure 17 shows the turning results of fishtails with different stiffness at different frequencies. From the turning comparison experiment in the figure, we can more intuitively obtain the same conclusion as the experimental data. In addition, the experimental results of turning radius are more directly expressed in Table 3.

## 5. Conclusions and Future Works

Inspired by the crank rocker, this paper designs a transmission mechanism called a double sine mechanism. Compared with the traditional wire-driven robotic fish, the mechanism designed in this paper can withstand greater lateral forces and ensure the stability of the robotic fish when swimming at high frequencies, with a maximum frequency of more than 7 Hz. The tail connector of the robotic fish uses torsion springs with three different diameters to simulate experiments under different tail stiffness. The experimental results show the following:

- As the tail swing frequency changes, the swimming speed change trends of the three groups of robotic fish with different tail stiffness are consistent. When the tail swing frequency is lower than 4 Hz, the linear swimming speed of the robotic fish increases with the increase in frequency, and when the tail swing frequency exceeds 4 Hz, its linear swimming speed decreases with the increase in frequency. When the frequency exceeds 7 Hz, the tail material reaches its limit and cannot swim normally.
- When the tail swing frequency is lower than 4 Hz, the swimming speed of the robotic fish with tail stiffness of 0.8 and 1.0 (the diameters of the torsion spring of the tail connector are 0.8 mm and 1.0 mm) gradually becomes weaker than that of the robotic fish with full rigid tail. This is because as the swing frequency increases, the response of the passive section of the tail with too low stiffness also slows down, thereby affecting the swimming speed.
- When the tail swing frequency exceeds 5 Hz, although the overall swimming speed tends to decrease, the swimming speed of the robotic fish with a tail stiffness of 1.0 is better than that of the robotic fish with a fully rigid tail. This is because when the frequency is too high, the active section of the tail cannot respond quickly, causing the fully rigid tail to be subject to greater water resistance. Due to the characteristics of the torsion spring, the passive section of the tail of the robotic fish with a tail stiffness of 1.0 will continue to swing outward for a distance in the original swing direction under the action of inertia, increasing its swing angle and reducing the influence of water resistance. Therefore, the swimming speed decreases relatively less.
- The turning radius of the robotic fish increases with the increase in frequency, and the weaker the tail stiffness, the larger the turning radius. This is because the lower the tail stiffness during turning, the easier it is to sweep a larger turning angle, resulting in a larger swimming radius. High-frequency swinging increases the swimming speed of the robotic fish, which also leads to an increase in the turning radius.

Although this study reveals the effect of different tail stiffness on swimming speed and agility under high-frequency motion, there is still a long way to go to catch up with real fish, including the turning mechanism, and there is still a gap compared with the turning of traditional servo-driven robotic fish. Future research will focus on the turning of the robotic fish in high-frequency mode.

## Figures and Tables

**Table 1:** Specifications of the robotic fish prototype.

	Size (mm)
Overall length of the robotic fish	430 × 102 × 140 (Length × Width × Height)
Head length	201
Body length	129
Tail length	100

**Table 2:** Parameter ratio of high-speed transmission mechanism.

Parameter	Actual Value	Parameter Ratio
r : R	1 cm:2 cm	1:2
m : M		1:6
l : d	1.32 cm:1.76 cm	3:4

**Table 3:** Radius of robotic fish with different tail stiffness at different frequencies.

	Frequency	1.0 Hz	1.5 Hz	2.0 Hz	2.5 Hz	3.0 Hz
Tail Stiffness						
0.8 mm		35.48 cm	49.04 cm	52.34 cm	8.48 cm	54.99 cm
1.0 mm		32.13 cm	32.36 cm	36.30 cm	37.54 cm	40.13 cm
Full rigid		21.75 cm	22.46 cm	24.06 cm	25.23 cm	27.98 cm

## Data Availability

No new data were created or analyzed in this study. Data sharing is not applicable to this article.
